# Characteristics of BRCA1/2 pathogenic germline mutations in chinese NSCLC patients and a comparison with HBOC

**DOI:** 10.1186/s13053-021-00174-1

**Published:** 2021-02-09

**Authors:** Zheyuan Xu, Yang Wang, Lan Wang, Fengxian Cui, Libin Zhang, Jian Xiong, Hao Peng

**Affiliations:** 1 Department of Thoracic Surgery, The First People’s Hospital of Yunnan Province, the Affiliated Hospital of Kunming University of Science and Technology, No. 157 Jinbi Road, 650032 Kunming, Yunnan, China; 2 Department of Anesthesiology, The First People’s Hospital of Yunnan Province, The Affiliated Hospital of Kunming University of Science and Technology, No. 157 Jinbi Road, 650032 Kunming, Yunnan, China

**Keywords:** Lung cancer, HBOC, BRCA1, BRCA2, Germline, Frameshift, Nonsense

## Abstract

**Background and purposes:**

The pathogenic BRCA1/2 germline mutations contributed to Hereditary Breast and Ovarian Cancer (HBOC) susceptibility. The features of BRCA1/2 germline mutations in non-small cell lung cancer (NSCLC) have not been systematically studied. Here we performed the first study investigating the characteristics of pathogenic BRCA1/2 germline mutations in Chinese NSCLC patients and compared them with those from Chinese HBOC.

**Methods:**

Information on BRCA1/2 germline mutations from 9010 Chinese NSCLC patients were collected from available studies and analyzed, and compared with the BRCA1/2 germline mutations from Chinese HBOC BRCA1/2 database (LOVD database, 20,523 patients).

**Results:**

19 (20 carriers, 0.22 %) pathogenic BRCA1 and 60 (66 carriers, 0.73 %) pathogenic BRCA2 germline mutations from NSCLC were identified. The carrier frequency of BRCA1/2 in Chinese NSCLC patients (86/9010 = 0.95 %) was significantly lower than that in Chinese breast and ovary cancer patients (1481/20,523 = 7.2 %) (P < 0.001). We found that frameshift and nonsense mutations were the predominant types of BRCA1/2 mutation in NSCLC, with no obvious hot spot mutations. No significant difference in the ratio of frameshift and nonsense mutations was found between BRCA1 and BRCA2 in NSCLC. 5 out of 19 mutations in BRCA1 and 23 out of 60 mutations in BRCA2 were novel mutations found in NSCLC that have never been reported in Chinese HBOC. A trend of higher percentage of BRCA1 nonsense mutations in the carriers was revealed in NSCLC compared with HBOC, while no such difference was found in BRCA2 in all types of mutations.

**Conclusions:**

BRCA1/2 germline mutations from NSCLC exhibited distinct characteristics compared with those from HBOC in Chinese population, including lower carrier frequency than HBOC, higher ratio of nonsense mutations and carriers than HBOC, and novel BRCA1/2 germline mutations never found in HBOC.

## Introduction

The germline mutations of BRCA1/2 have been comprehensively investigated in Hereditary Breast and Ovarian Cancer (HBOC). The interpretation of pathogenicity of BRCA1/2 germline mutations is based on the American College of Medical Genetics (ACMG) guidelines and provides strong tools for new mutation identification and interpretation [1]. In terms of pathogenicity, most BRCA1/2 mutations are sporadic mutations with no pathogenic nature, and these mutations are classified as benign, likely benign or VUS (variants of uncertain significance) [1]. Only pathogenic and likely pathogenic mutations are actionable and patients with these mutations need to be treated actively. PARP inhibitors have been widely used for the treatment of HBOC at first and multiple line levels with good response and compliance [2].

Apart from HBOC, the germline BRCA1/2 mutations have also been found in a variety of other cancers, including pancreatic cancer [3], lung cancer [4], urothelial carcinoma [5] and esophageal cancer [6], etc. It is reasonable to expect that PARPi may also be effective for these patients. However, the evidence for the effectiveness of PARPi treatment on these cancers is limited so far. This is because the incidence of BRCA1/2 germline mutations in these cancers is much lower than that in HBOC [7], making it difficult to perform large-scale clinical trials. In this study, we focused on the characteristics of BRCA1/2 germline mutations in NSCLC, and compared the features of these mutations with those found in HBOC. We identified distinct mutational characteristics and novel germline mutations of BRCA1/2 in NSCLC, providing useful information for potential PARPi treatment of NSCLC with such mutations.

## Methods and materials

### Data collection, analysis and interpretation of BRCA1/2 mutations in NSCLC

The information on mutations and specific mutation sites were collected from three publications reporting the BRCA1/2 germline mutations in Chinese population [8–10]. Mutation sites were reorganized in protein change format and the carrier frequency was calculated for BRCA1 (Table 1) and BRCA2 (Table 2). The pathogenicity of all germline mutations were interpreted based on the guidelines for variant classification jointly issued and revised by the American College of Medical Genetics (ACMG) and the Association for Molecular Pathology (AMP) [1]. Only mutations interpreted as pathogenic were included for analysis in this study, and mutations with other pathogenicity (likely pathogenic, VUS, likely benign and benign) were excluded.

**Table 1:** The details of germline BRCA1 mutations and the number of carriers for lung cancer patients

Mutations	Mutation Type	Number of carriers in Lung Cancer	Number of carriers in Breast and Ovary Cancer
I1824Dfs*3	Frameshift	2	134
Exon4-splice	Splice site variant	1	13
R1443*	Nonsense	1	11
E489*	Nonsense	1	9
E1158*	Nonsense	1	8
L502Afs*2	Frameshift	1	4
E720*	Nonsense	1	3
S1503*	Nonsense	1	2
Exon3-splice	Splice site variant	1	1
H318Lfs*24	Frameshift	1	1
T715fs	Frameshift	1	1
L1209*	Nonsense	1	1
F1571fs	Frameshift	1	1
G1743fs	Frameshift	1	1
K339Rfs*2	Frameshift	1	0
E755fs	Frameshift	1	0
Q934*	Nonsense	1	0
K1079*	Nonsense	1	0
Y1666*	Nonsense	1	0

**Table 2:** The details of germline BRCA2 mutations and the number of carriers for lung cancer patients

Mutations	Mutation Type	Number of carriers in Lung Cancer	Number of carriers in Breast and Ovary Cancer
Q1037*	Nonsense	3	48
S1722fs	Frameshift	2	25
K936fs	Frameshift	1	16
F2801fs	Frameshift	1	16
K157Sfs*24	Frameshift	1	12
A938fs	Frameshift	1	12
N3024fs	Frameshift	1	10
M815fs	Frameshift	1	7
S1900*	Nonsense	1	7
N433fs	Frameshift	1	6
R2336L	Missense	2	6
N2135fs	Frameshift	1	5
T3033fs	Frameshift	1	5
S2120*	Nonsense	1	4
R2318*	Nonsense	1	4
I591fs	Frameshift	1	3
Q1987*	Nonsense	1	3
Exon24-splice	Splice site variant	1	3
I605fs	Frameshift	1	2
S780*	Nonsense	1	2
L929*	Nonsense	1	2
Y949fs	Frameshift	1	2
M2235fs	Frameshift	1	2
S2984*	Nonsense	1	2
G3134Afs*29	Frameshift	1	2
D252fs	Frameshift	2	1
P1145*	Nonsense	1	1
L1908fs	Frameshift	1	1
N2137fs	Frameshift	1	1
Exon16-splice	Splice site variant	2	1
Q2530*	Nonsense	1	1
L2573*	Nonsense	1	1
exon21-splice	Splice site variant	1	1
8487+1G>C	Splice site variant	1	1
K3004fs	Frameshift	1	1
R3052W	Missense	1	1
M3118fs	Frameshift	1	1
S158*	Nonsense	1	0
D306fs	Frameshift	1	0
E357*	Nonsense	1	0
T598fs	Frameshift	1	0
E790*	Nonsense	1	0
Y828*	Nonsense	1	0
N1055fs	Frameshift	1	0
D1210fs	Frameshift	1	0
K1239fs	Frameshift	1	0
D1476fs	Frameshift	1	0
I1485Nfs*3	Frameshift	1	0
L1616fs	Frameshift	1	0
E1625fs	Frameshift	1	0
E1646Nfs*19	Frameshift	1	0
Y1661*	Nonsense	1	0
V1681fs	Frameshift	1	0
R1694*	nonsense	1	0
K1964*	Nonsense	1	0
H1966fs	Frameshift	1	0
I2149fs	Frameshift	1	0
G2274fs	Frameshift	1	0
R2896fs	Frameshift	1	0
E3258fs	Frameshift	1	0

### Data processing and analysis from the LOVD database

The information on mutations and specific mutation sites was downloaded from the LOVD database following the link below:

https://databases.lovd.nl/shared/variants?search_owned_by_=%3D%22Xianqi%20Gao%22.

The set of data was reported in a recent publication comprehensively reviewed and summarized the BRCA1/2 germline mutations from HBOC in Chinese population from 1999 to 2017 [11]. Therefore, the database represents a full collection of Chinese BRCA1/2 germline mutations so far. The BRCA1/2 mutations found in NSCLC were searched in the LOVD database, and the corresponding carrier frequency in HBOC was determined and compared with that from NSCLC (Tables 1 and 2). The mutational frequency, carrier frequency, and mutational type information for HBOC was also collected and summarized for analysis.

### Statistics and figure plot

Statistical analysis was performed, and figures were plotted with GraphPad Prism 5.0 software (GraphPad Software, Inc, La Jolla, CA 92,037, USA) or the R software (https://www.r-project.org/). Chi-square test and Fisher test were performed when rate or percentage was compared for significance. P < 0.05 was regarded as significant difference.

## Results

### Main features of the pathogenic BRCA1/2 germline mutations in NSCLC patients

**Fig. 1:**
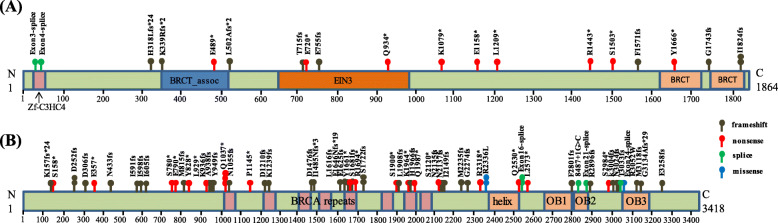
Scheme shows the position, distribution and types of all BRCA1/2 pathogenic germline mutations revealed in recent studies in Chinese population. Mutations of BRCA1 (panel **a**) and BRCA2 (panel **b**) are shown, respectively, with key domains labeled

The information for all pathogenic BRCA1/2 mutations found in NSCLC was collected and analyzed. All mutations were plotted in a scheme to show the distribution and types of mutations (Fig. 1). 19 (20 carriers) pathogenic BRCA1 (Fig. 1A) and 60 (66 carriers) pathogenic BRCA2 (Fig. 1B) germline mutations were identified. It can be observed that frameshift and nonsense mutations were the predominant types of BRCA1/2 mutation in NSCLC. 8 out 19 mutations were frameshift and 9 out of 19 mutations were nonsense in BRCA1 (Table 1). 36 out of 60 mutations were frameshift and 18 out of 60 mutations were nonsense in BRCA2 (Table 2). No significant difference in the ratio of frameshift and nonsense mutations was found between BRCA1 and BRCA2 in NSCLC. The distribution of the mutations appeared to be sporadic in both BRCA1 and BRCA2, and mutations can be found inside and outside the key functional domains, and there were no obvious hot spot mutations. The percentage of BRCA1/2 germline mutation carriers in Chinese NSCLC patients (86/9010 = 0.95 %) was significantly lower than that in Chinese breast and ovary cancer patients (1481/20,523 = 7.2 %) (P < 0.001), suggesting a cancer-type dependent incidence of BRCA1/2 germline mutations.

### Comparison of BRCA1/2 germline mutation characteristics between NSCLC and HBOC

**Fig. 2:**
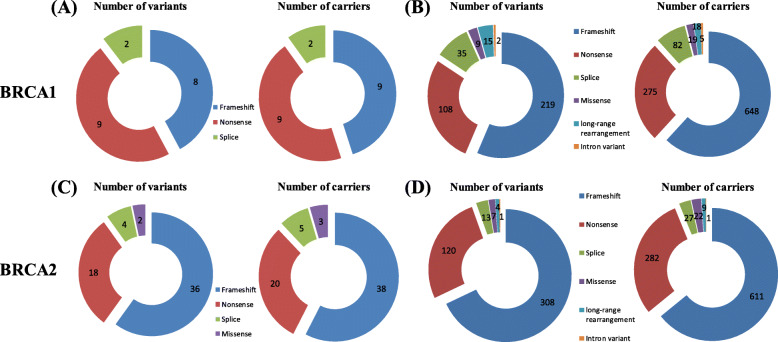
The numbers and relative ratio for each type of BRCA1/2 germline mutation in NSCLC and HBOC. Panel **a** and **b** show the numbers, ratio for various types of BRCA1 mutations in NSCLC (**a**) and HBOC (**b**), respectively. Panel **c**and **d** show the numbers, ratio for various types of BRCA2 mutations in NSCLC (**c**) and HBOC (**d**), respectively. Mutation types are shown by different colors as labeled

BRCA1/2 mutational information of HBOC from the LOVD database was summarized and the mutational features were compared with those from NSCLC. The number of variants or carriers and the percentage of BRCA1/2 in NSCLC and HBOC are shown in Fig. 2. Potential higher percentage of BRCA1 nonsense mutations and the carriers was revealed in NSCLC (Fig. 2A) compared with HBOC (Fig. 2B) (Chi-square test, P = 0.069 for variant percentage and P = 0.060 for carrier percentage). In contrast, no such difference was found in BRCA2 in all types of mutations between NSCLC (Fig. 2 C) and HBOC (Fig. 2D).

**Fig. 3:**
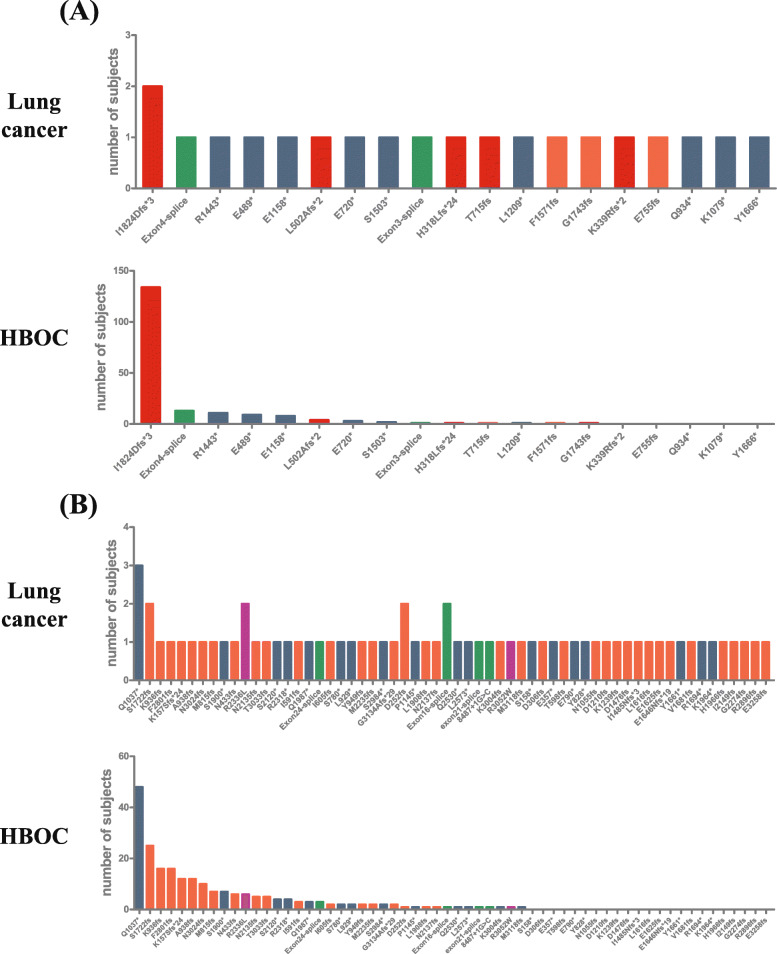
The numbers of carriers for each BRCA1/2 mutations in NSCLC and corresponding numbers of carriers in HBOC. Panel **a** shows the number of carriers for BRCA1 mutations in NSCLC (upper panel) and the carriers for these mutations in HBOC (lower panel). Panel **b** shows the number of carriers for BRCA2 mutations in NSCLC (upper panel) and the carriers for these mutations in HBOC (lower panel)

The individual mutations found in NSCLC were compared with those in HBOC (Fig. 3). It was revealed that 5 out of 19 pathogenic mutations in BRCA1 (Fig. 3A; Table 1) and 23 out of 60 pathogenic mutations in BRCA2 (Fig. 3B; Table 2) were novel mutations found in NSCLC that have never been reported in Chinese HBOC. These novel mutations were all low-frequency mutations in NSCLC, suggesting their sporadic nature.

## Discussion

The BRCA1/2 germline mutations in HBOC have been widely studied in many reports, and databases such as BIC, ClinVar, BRCA Share and ENIGMA, collected huge number of BRCA1/2 germline mutations [11, 12]. However, the mutations recorded by these databases are mainly from Caucasian, black and Jewish populations. It was not until very recently that the database for BRCA1/2 germline mutations in Chinese population was established [11]. Studies so far found that BRCA1/2 germline mutations varied substantially across different populations. Unlike other hereditary cancer syndrome such as Lynch syndrome, no definite hot spot BRCA1/2 mutations were found in several populations. However, definite hot spot mutations were observed in Ashkenazi Jewish population, exhibiting clear founder effect [11]. Reports in Chinese population did not find founder effect, although some high frequency mutations were observed [11]. This could be due to the fact that Jewish population had relatively strict endogamy while large-scale population migration across east and central Asia happened in Chinese population Therefore, studies on BRCA1/2 germline mutations must consider the discrepancy across different races. The panorama of BRCA1/2 mutations in Chinese was recently established to facilitate the diagnosis and therapy in the population.

BRCA1/2 germline mutations were not only reported in HBOC, but also reported in many other cancer types, including pancreatic cancer [3], lung cancer [4], urothelial carcinoma [5] and esophageal cancer [6]. The frequency of BRCA 1/2 germline mutations in these cancer appeared to be significantly lower than that in HBOC, showing clear HBOC preference. For example, a study involving 854 patients with pancreatic ductal adenocarcinoma showed that 12 patients (1.4 %) had BRCA2 and 3 patients (0.35 %) had BRCA1 deleterious germline mutations [13]. Our study on Chinese NSCLC patients found 20 patients (0.22 %) with BRCA1 and 66 patients (0.73 %) with BRCA2 pathogenic germline mutations, representing a comprehensive report on the incidence of BRCA1/2 germline mutations in Chinese NSCLC. This incidence was much lower than that reported in Chinese HBOC, in which 5.74 % of breast cancer and 21.79 % of ovarian cancer patients had BRCA1/2 germline mutations, with an overall pathogenic mutation frequency of 69.9 % and 71.1 % for BRCA1 and BRCA2 exons, respectively [11]. Indeed, studies have suggested that no significant difference was found in the ratio of NSCLC patients with smoking history between those with or without pathogenic germline mutations [8, 10], and germline mutations in driver oncogenes and inherited lung cancer risk may be independent of smoking history [14]. These observations suggested that the influence of BRCA1/2 germline mutations in NSCLC may not be as big as that in HBOC, and factors other than germline variants may play more important roles in sporadic lung cancer patients, most likely environmental factors including smoking [10].

We found that most pathogenic mutations in BRCA1/2 in NSCLC were frameshift and nonsense mutation. Indeed, these mutations may cause large fragment aberrancies and lead to protein dysfunction, which explains their pathogenic nature. Similar trend was also found in HBOC, in which frameshift appeared to be the mutation type with highest ratio in both BRCA1 and BRCA2 [11]. In contrast, we found that the BRCA1 nonsense mutations in NSCLC showed a trend of higher ratio than that in HBOC. If this was real, the observation suggested a preference of nonsence mutation in BRCA1 in NSCLC, however, this could be affected by the limited number of mutations in BRCA1 in NSCLC, as only a total of 20 patients were involved. No significant difference in the ratio of frameshift and nonsense mutations was found in BRCA2 between NSCLC and HBOC, suggesting a similar mutation type distribution across different cancers. This observation also indicated that the sporadic distribution and no-hot-spot nature of BRCA1/2 mutations may exist in all cancers and possibly in general population, while the difference in BRCA1/2 incidence across cancer types may be due to the distinct enrichment effect of BRCA1/2 mutations in different cancers.

An interesting observation in this study is that family history of lung cancer has been seen in 57.14 % (8/14) of lung cancer patients with pathogenic germline mutation, and in 32.35 % (11/34) of patients with likely pathogenic germline mutations [10]. Therefore, it can be suggested that NSCLC did happen to a family when pathogenic germline mutations were inherited. Meanwhile, the same study also reported that multiple cancer types were found in 21.43 % (3/14) of patients with pathogenic germline mutations, and in 11.76 % (4/34) of patients with likely pathogenic germline mutations, suggesting that pathogenic or likely pathogenic germline mutations may also increase the risk of other cancers besides lung cancer. This was also reported in a couple of case studies. Boettger et al. found a lung cancer patient with BRCA2 pathogenic germline mutation with strong family history of breast cancer [15], and Marafie et al. also reported a lung cancer patient from an extended family segregating different types of hereditary cancer over several generations, including lung, breast, ovarian, colon, prostate and renal cancers [16]. Therefore, it can be suggested that the emergence of cancers in these families was not due to the co-finding of pathogenic germline mutations with environmental factor-relevant cancers but was correlated with the risk from germline mutations.

In this study, we also identified several novel pathogenic germline mutations in NSCLC that have never been found in Chinese HBOC patients. New germline mutations of BRCA1/2 have been emerging all the time in the past 20 years in HBOC [11]. It appeared that the sporadic nature of the BRCA1/2 mutations may continuously generate novel mutations, not only in HBOC, but also in NSCLC. Therefore, the novel mutations found in NSCLC may not be cancer-specific, but related to population-based sporadic distribution. If the examined population is large enough, more novel mutations may be revealed. It was interesting in this study that a similar trend of mutational frequency may exist between NSCLC and HBOC. For example, BRCA2 Q1037* was the mutation with highest frequency in both NSCLC and HBOC. Although the number of patients was limited in NSCLC, this trend could be a reflection of mutational spectrum similarity in Chinese population across different cancers. Therefore, we speculate that the background BRCA1/2 mutational status in cancers may be similar, and the emergence of certain mutations in a cancer may be random, while the enrichment of the mutations may be cancer-specific.

PARP inhibitors (PARPi) have been widely used in the treatment of HBOC, and their effectiveness have been proved by many studies at first-line and multiline levels [17, 18]. Maintenance therapy with PARPi has been approved for HBOC therapy in patients who are sensitive to platinum-based chemotherapy, which plays a key role and has been applied in the first-line therapy of ovarian and breast cancers for more than two decades [19]. Although various combinations with platinum-based reagents have been investigated over the years to find the best strategy for optimal response and minimal toxicity, it seems difficult to further improve the response while balance the toxicity of these drugs [20–22]. Maintenance therapy was therefore developed to solve the issue in patients who achieved an initial response to chemotherapy with the goal to maintain the response with an acceptable toxicity [23]. Therefore, platinum-based chemotherapy is still important for initial therapy of these patients, and PARPi has crucial roles in maintaining the response and reducing the side effects.

Advances have also been made in the application of PARPi in cancers other than HBOC with BRCA1/2 germline or somatic mutations. For example, for metastatic pancreatic cancer patients with germline BRCA mutations who had not progressed during first-line platinum-based chemotherapy, progression-free survival was longer with maintenance Olaparib than with placebo [24]. There are also a couple of ongoing clinical trials designed to evaluate the safety and efficacy of PARPi in urothelial carcinoma as monotherapy or in combination with other drugs, including two ongoing phase II clinical trials (NCTXXXX and NCTXXXX), but specific efficacy data have not been reported [25]. In contrast, no clinical trial data is available so far for NSCLC treatment with PARPi, possibly due to the plenty of therapeutic methods for NSCLC and the very low incidence of BRCA1/2 germline mutations in NSCLC.

In conclusion, our study systematically investigated the characteristics of BRCA1/2 germline mutations in Chinese NSCLC patients, and compared the features of the mutations with those from Chinese HBOC. We found that BRCA1/2 germline mutations in NSCLC had lower carrier frequency than HBOC, potential higher ratio of BRCA1 nonsense mutations and carriers than HBOC, and revealed several novel BRCA1/2 germline mutations that have never been reported in Chinese HBOC.

## Data Availability

The data that support the findings of this study are openly available in LOVD at https://databases.lovd.nl/shared/variants?search_owned_by_=%3D%22Xianqi%20Gao%22. Other data are available from the corresponding author upon reasonable request.
